# First case report of anamorelin-induced fatal arrhythmia complicated by sinus arrest and refractory ventricular tachycardia

**DOI:** 10.1016/j.hrcr.2022.12.011

**Published:** 2022-12-14

**Authors:** Keisuke Kojima, Shoichiro Furukawa, Tomoyuki Ishikawa, Shujiro Inoue

**Affiliations:** Department of Cardiology, Aso Iizuka Hospital, Iizuka, Japan

**Keywords:** Anamorelin, Drug-induced arrhythmia, Ventricular tachycardia, Sick sinus syndrome, Sodium channel

## Introduction

Anamorelin is the first drug for the treatment of cancer cachexia. It is an oral small-molecule ghrelin-like drug that stimulates growth hormone secretion and appetite.^1^ In clinical trials in patients with inoperable stage III or IV non–small cell lung cancer and cachexia, it improved appetite scores and increased lean body mass, a measure of the skeletal muscle mass.^2^^,^^3^ Moreover, this drug has been noted for its proarrhythmic effects by decreasing sodium channel currents and L-type calcium channel currents; however, there are few reports on the detailed mechanisms and arrhythmias. Here we report the first case of fatal arrhythmia complicated by sinus arrest and refractory ventricular tachycardia induced by a single dose of this drug.

## Case report

An 84-year-old woman with stage IV rectal cancer presented to our emergency department with a complaint of sudden fatigue. The patient underwent surgery for rectal cancer 5 years before. She was diagnosed with lymph node and lung metastases after surgery and placed on radiochemotherapy (bevacizumab, TS-1) for 2 years. She had had treatment-induced anorexia. Her electrocardiogram findings were normal, and anamorelin was prescribed for cancerous cachexia.

She took anamorelin (100 mg) for the first time on the morning of her visit. Approximately 3 hours later, she suddenly became very tired and laid down in bed. Her neighbor realized that the patient had difficulty in moving around and called an ambulance to take her to the emergency department.

**Figure 1:**
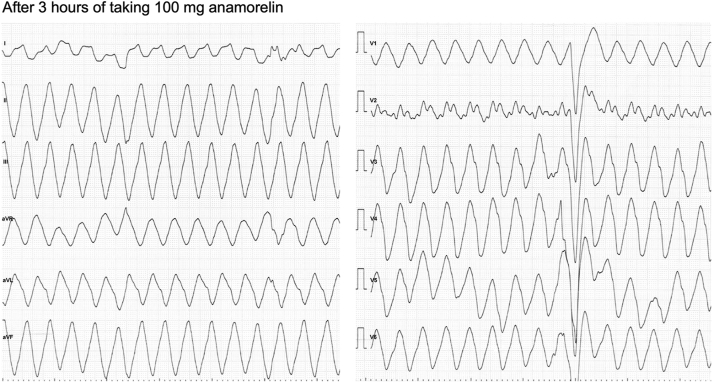
Electrocardiogram at presentation. Monomorphic ventricular tachycardia (wide QRS complex; 280 ms) at a rate of 202 beats/min.

The ambulance monitor electrocardiography revealed wide QRS tachycardia, and a 12-lead electrocardiogram on arrival (Figure 1) revealed heart rate 202 beats per minute and monomorphic ventricular tachycardia (VT) with a marked wide QRS complex (280 ms). She was conscious but looked weak and pale. On arrival, her physical examination revealed peripheral coldness of her limbs and a blood pressure of 90/52 mm Hg. Her respiratory rate was 32 cycles per minute. Blood chemistry showed no signs of liver or kidney impairment and no obvious electrolyte imbalances. Markedly elevated lactate and metabolic acidosis (pH, 7.14; lactate, 104.9 mg/dL) showed circulatory failure.

**Figure 2:**
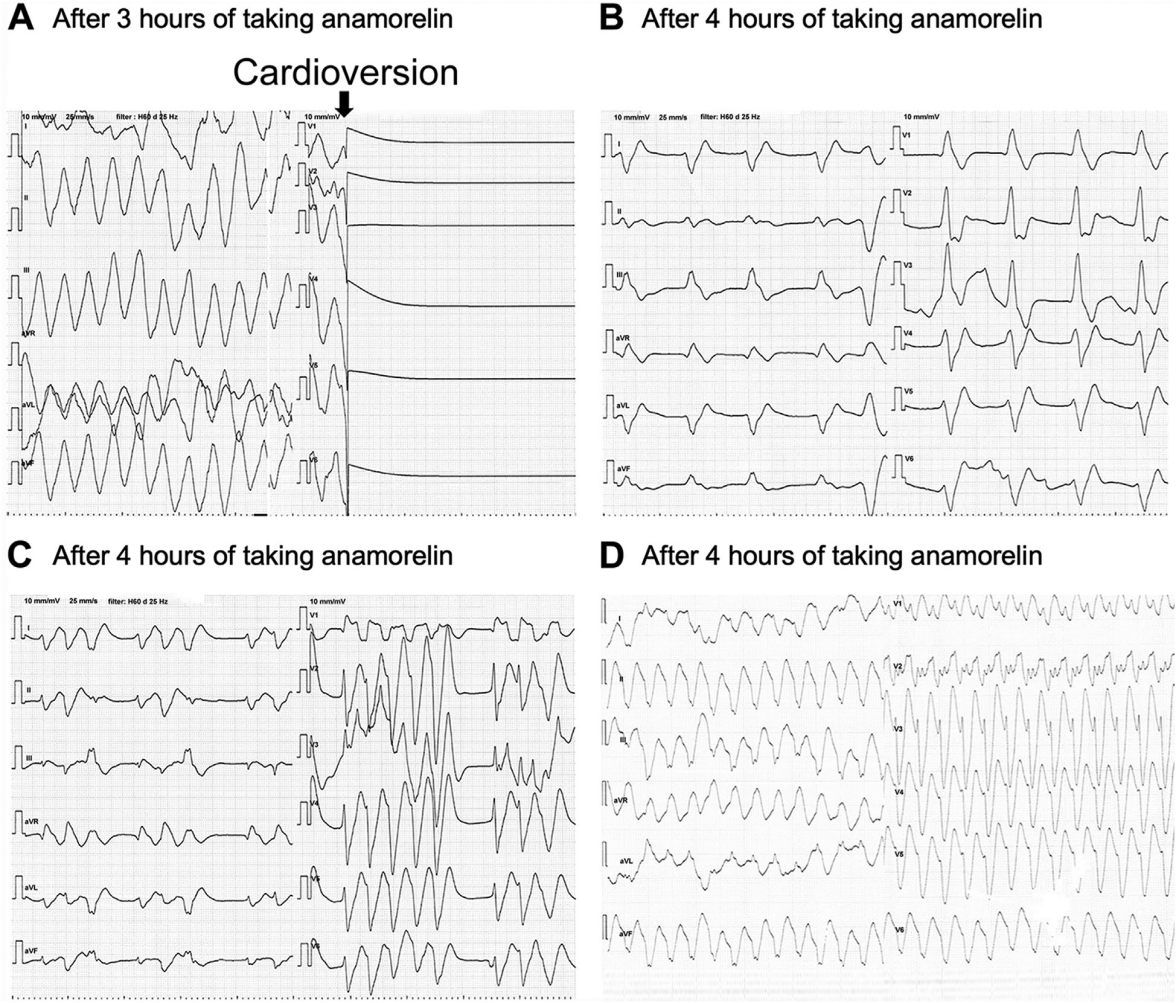
Electrogram before treatment. **A:** Sinus arrest appeared after defibrillation; thus, we immediately performed cardiopulmonary resuscitation. **B, C:** After spontaneous circulation, the electrocardiogram finding changed to a ventricular escape rhythm (**B**) and an escape rhythm with incessant polymorphic ventricular tachycardia (**C**).

The patient was diagnosed with hemodynamically unstable VT and underwent synchronized defibrillation at 100 J of energy. VT stopped but cardiac arrest soon followed (Figure 2A). Chest compressions were started immediately. After 1 cardiopulmonary resuscitation cycle, the patient recovered ventricular escape rhythm (Figure 2B) and repeated continuous polymorphic VT (Figure 2C) or monomorphic VT (Figure 2D).

The patient was moved while undergoing percutaneous external pacing, and an external pacemaker was urgently placed for sinus arrest. Because the bradycardia was backed up by pacing, we tried several antiarrhythmic agents for VT. The administration of both 10 mg of adenosine 5'-triphosphate and 40 mg of lidocaine did not suppress the incessant VT. However, the intravenous administration of amiodarone at a dose of 75 mg suppressed this VT.

Coronary angiography was performed but revealed no significant stenosis. Left ventriculography revealed mild hypokinesis only at the apex. At this time, we were unable to identify the cause of the simultaneous onset of severe bradycardia and versatile VT.

**Figure 3:**
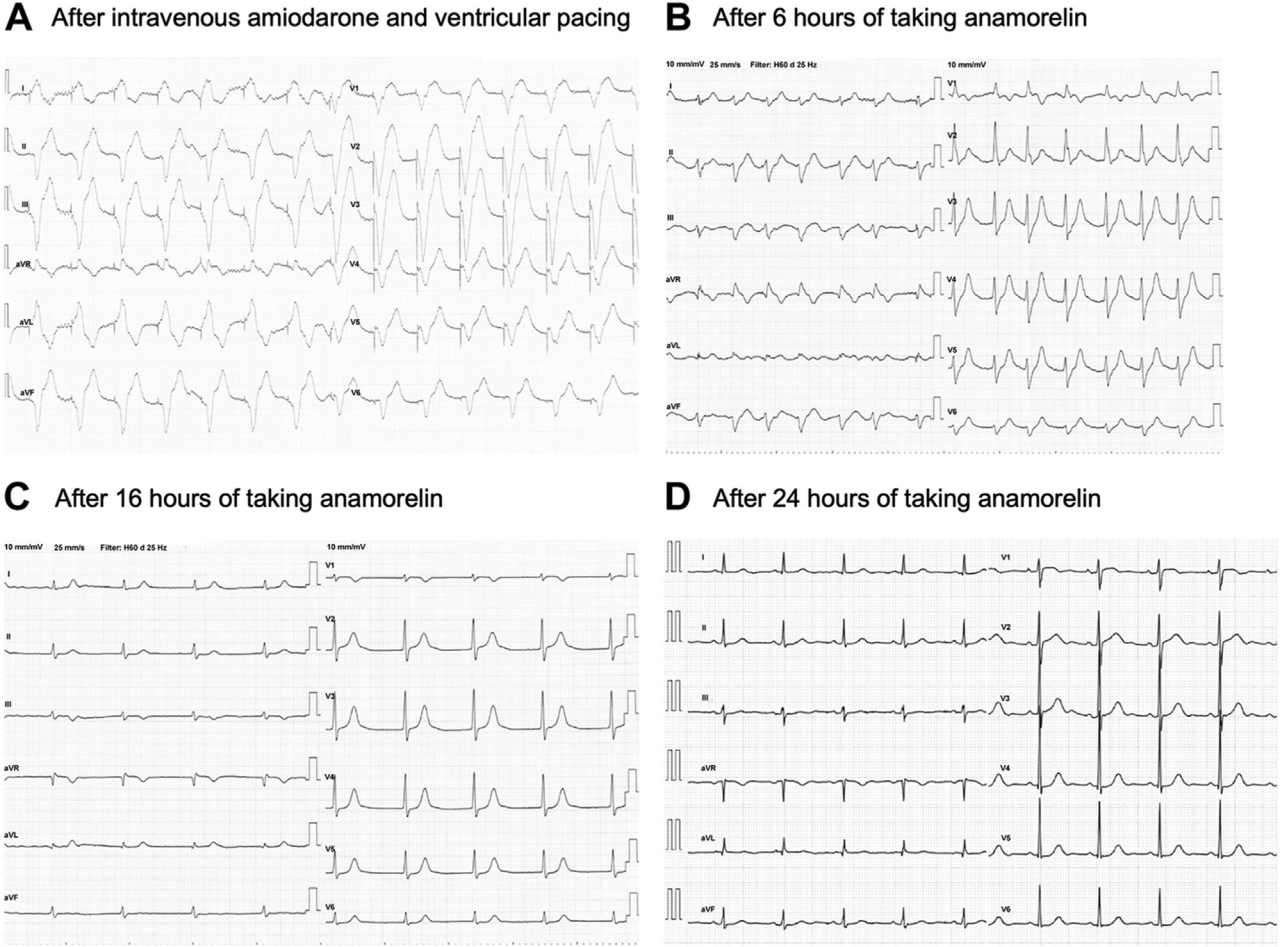
Electrogram after treatment (amiodarone) and drug discontinuation. **A:** After admission, ventricular tachycardia was suppressed using intravenous amiodarone, and an idioventricular rhythm appeared. **B:** After 6 hours of taking anamorelin, the QRS complex of the ventricular escape became narrow. **C:** After 16 hours of taking anamorelin, an atrioventricular junction rhythm was maintained. **D:** After 24 hours of taking anamorelin, a P wave appeared, and the patient demonstrated a sinus rhythm.

Amiodarone was judged to be effective in suppressing VT, and continuous amiodarone administration (17 mL/h) was started while backing up bradycardia with pacing (Figure 3A). The examination of the patient’s oral medications revealed that anamorelin had been started the day she was admitted for anorexia, and that the drug had proarrhythmic effects, such as sodium channel blockade. Therefore, these were possibly drug-induced arrhythmias, and anamorelin was discontinued thereafter. After returning to the ward, the patient was in a ventricular pacing waveform (heart rate 80 beats/min), and VT was observed in a nonsustained form; however, shortly after, VT was no longer observed. A ventricular rhythm was then observed (Figure 3B), and when pacing was stopped 12 hours after admission, the patient was in atrioventricular junction rhythm (Figure 3C). Approximately after 24 hours of taking anamorelin, the P wave appeared, and normal sinus rhythm was restored (Figure 3D). Thereafter, further arrhythmia was never observed, and the temporary pacemaker was removed the next day. Electrocardiography and echocardiography revealed no evidence of organic heart disease, and the patient had a reversible course; thus, we concluded that the arrhythmia was drug-induced (by anamorelin).

The patient was transferred to the department of palliative care for continued rehabilitation. Approximately 6 months after admission, the patient died of colorectal cancer–related bowel obstruction; however, no cardiac event was observed after this drug-induced arrhythmia.

## Discussion

The course of this patient demonstrates 2 important clinical implications.

First, anamorelin can cause life-threatening arrhythmias; thus, careful monitoring is required before and after its administration. The package insert specifies that the drug should be administered with caution because of its inhibitory effects on the conduction system owing to sodium channel blockade. Moreover, the electrocardiogram and pulse should be regularly monitored before and during administration, and if any abnormality is observed, the administration should be discontinued, especially during the early stages of administration. The patient developed various forms of VT and sinus arrests several hours after receiving a new single dose of anamorelin. Two case reports of VT associated with this drug exist; in these cases the patients showed a similar electrocardiogram waveform and course to our patient in this case report.^4^^,^^5^ However, as far as we know, this is the first case of simultaneous-onset VT and sinus arrest. Amiodarone was remarkably effective for this VT, as in the case reported in the past.^4^ A 100 mg oral dose of anamorelin peaks at 0.75 hours, and the half-life is 8.8 hours. In this patient, symptoms abruptly occurred approximately 3 hours after a single dose and the patient returned to normal sinus rhythm approximately 20 hours after its discontinuation. We concluded that the pharmacokinetics of anamorelin did not contradict the clinical course of our patient. It has been used in 32 patients with cancer-related cachexia at our institution. Among them, adverse events of reversible arrhythmia occurred in 3 cases (1 case of complete atrioventricular block, 1 case of first-degree atrioventricular block, and 1 case of complete right bundle branch block). As there were no abnormalities in the electrocardiogram and echocardiography before its administration in this patient, it was considered essential to observe the electrocardiogram before and after administration in all cases treated with anamorelin.

Second, the mechanism of the proarrhythmic properties of anamorelin is discussed. Although the mechanism of anamorelin’s proarrhythmic action has not been fully elucidated, weak binding to sodium channels and L-type calcium channels has been observed.^6^ Decreased sodium currents may predispose patients to sudden cardiac death, as the cardiac arrhythmia suppression test revealed that sodium channel blockers increase the incidence of sudden cardiac death.^7^ In particular, conduction disturbances due to sodium channel blockade may cause reentrant arrhythmias owing to the enlarged excitable gap.^8^ In addition, sinus arrest in this patient might have been influenced by the effect on L-type calcium channels.^3^ In this patient, severe fatal arrhythmia developed after a single oral dose of the prescribed medication. For this drug, elevated blood concentrations have been reported in patients with severe hepatic dysfunction. In particular, it is reported to be affected by the hepatic drug–metabolizing enzyme, CYP3A4, and drugs that inhibit CYP3A4 may have increased serum levels.^9^ This patient did not take drugs that have been shown to interact with CYP3A4, nor did he have severe liver dysfunction. However, the range of poisoning (side effects), such as electrocardiogram abnormalities and proarrhythmia, cannot be determined only by the blood concentration because factors other than the concentration of the antiarrhythmic drug, such as electrolytes and genetic factors, are also involved.

Reportedly, there are some patients who respond well to concentrations lower than the general effective blood concentration range and others who do not respond well even within that range.^10^ One reason for this may be that the effective blood concentration of sodium channel blockers required to exert antiarrhythmic effects may differ among individual patients owing to genetic differences in myocardial sodium channel function. For example, the *SCN5A* gene associated with Brugada syndrome encodes the alpha subunit of the cardiac sodium channel Nav1.5 and is responsible for phase 0 of the cardiac action potential.^11^ Six associated polymorphisms (haplotype; Hap) are known in the promoter region of the *SCN5A* gene. Among them, it has been pointed out that in patients with Hap B, sodium channel expression levels are reduced, and the effects of sodium channel blockers are stronger in such patients than in patients with wild-type gene. Hap B is unique to Asians, with a prevalence of 24% in Japanese people. Patients who respond to sodium channel blockers at low doses may have Hap B. It is possible that this patient also developed lethal arrhythmia owing to differences in reactivity due to sodium channel gene polymorphisms.

The polymorphism of the cardiac sodium channel was not analyzed in this patient, which is a limitation of our study. It has been suggested that anamorelin’s effects on sodium channel receptors are generally very low in the oral blood concentration range. In this patient, the high sodium channel sensitivity of this drug may have caused a strong sodium channel–blocking effect or the involvement of L-type calcium channels; however, the detailed mechanism is unclear.

## Conclusion

Anamorelin can cause fatal arrhythmias; thus, careful monitoring before and after its administration is required. The patient should fully understand the benefits and risks associated with anamorelin and consent to its use; moreover, considering the risk of side effects, it is preferable to start this drug during hospitalization. Its proarrhythmic effects have been suggested to be mediated by sodium channels and L-type calcium channel blockade. In addition to the blood concentration or dose dependence, sodium channel gene polymorphisms may be associated with the onset of the disease. Further reports and the elucidation of the mechanism involved are needed, and it is hoped that anamorelin can be used more safely.

**Key Teaching Points**

• Anamorelin can cause fatal arrhythmias; thus, careful monitoring is required before and after its administration.
• The proarrhythmic effects of anamorelin have been suggested to be mediated via sodium channels and L-type calcium channel blockade.
• Amiodarone may be effective for this ventricular tachycardia.
